# Ketamine as an adjunctive therapy for major depression - a randomised controlled pragmatic pilot trial (Karma-Dep Trial)

**DOI:** 10.12688/hrbopenres.13182.2

**Published:** 2022-01-27

**Authors:** Bronagh Gallagher, Meabh Foley, Claire M. Slattery, Gabriele Gusciute, Enda Shanahan, Declan M. McLoughlin

**Affiliations:** 1 Department of Psychiatry, Trinity College Dublin, St. Patrick’s University Hospital, James Street, Dublin, Ireland; 2 Institute of Neuroscience, Trinity College Dublin, Dublin, Ireland

**Keywords:** Ketamine, Depression, Adjunctive, Pilot trial

## Abstract

**Background**: Depression is a common psychiatric disorder that has become the leading cause of disability worldwide. The standard medical care for depression over the past 50 years has focused on monoamine neurotransmitters. These treatments can take weeks to take effect, highlighting the need for novel treatment strategies. One such approach may be ketamine. Ketamine acts as an antagonist of the N-methyl-D-asparate receptor and thus targets the excitatory amino acid neurotransmitter glutamate. Interestingly, at sub-anaesthetic doses, a single infusion of ketamine can elicit a rapid, though transient, antidepressant response.

**Methods**: The aim of this study was to conduct a pragmatic randomised controlled pilot trial of four once-weekly ketamine infusions as an adjunctive therapy for depression.  The main objective was to assess trial procedures to inform a future definitive trial. The primary clinical outcome was the 24-item Hamilton Rating Scale for Depression (HRSD-24). Trial participants were patients admitted to St Patrick’s Mental Health Services for treatment of a depressive episode. They underwent usual inpatient care as prescribed by their treating team. Consented participants were randomly allocated to a four-week course of either once-weekly ketamine (0.5mg/kg) or midazolam (0.045mg/kg) infusions given over 40 minutes and with 12 weeks follow-up.

**Results**: In total, 1581 admissions to St Patrick’s Hospital were assessed for eligibility over nine months, with 125 (8%) meeting criteria, with 25 (20%) providing consent. In total, 13 were randomly assigned to the ketamine arm and 12 to the midazolam arm. There were no major differences in HRSD-24 scores between the two groups. The infusions were generally safe and well tolerated.

**Conclusions**: This is the first pragmatic pilot trial of adjunctive serial ketamine infusions for hospitalised depression, an important possible use of ketamine. This study suggests that a definitive trial of adjunctive ketamine is feasible.

**Trial registration**: ClinicalTrials.gov NCT03256162 21/08/2017; EudraCT 2016-004764-18 30/11/2016.

## Abbreviations

BPRS; Brief Psychiatric Rating Scale; CADSS: Clinician-administered Dissociative States Scale; DSM-5: Diagnostic and Statistical Manual of Mental Disorders; Fifth edition; FDA: Food and Drug Administration; GCP: Good Clinical Practice; HPRA: Health Products Regulatory Authority; HRSD-11: Hamilton Rating Scale for Depression, 11-item; HRSD-24: Hamilton Rating Scale for Depression, 24-item; ICH: International Conference on Harmonisation; MADRS: Montgomery-Asberg Depression Rating Scale; MDD: Major Depressive Disorder; MINI: Mini International Neuropsychiatric Interview; MoCA: Montreal Cognitive Assessment; MSMTRD: Maudsley Staging Method for Treatment Resistance in Depression; NICE: National Institute for Health and Care Excellence; NMDA: N-methyl-D-asparate; PRISE: Patient-rated Inventory of Side-effects; QIDS-SR-16: Quick Inventory of Depressive Symptoms, Self-Report, 16-item; RCT: Randomised Controlled Trial; SNRI: Serotonin-Norepinephrine Reuptake Inhibitor; SSRI: Selective Serotonin Reuptake Inhibitors; STAR*D: Sequenced Treatment Alternatives to Relieve Depression; TRD: Treatment Resistant Depression; YMRS: Young Mania Rating Scale

## Introduction

Depression is a common psychiatric disorder that causes significant disability. It can seriously impair occupational and social functioning, resulting in reduced quality of life ^1^. According to the World Health Organisation, depression affects more than 300 million people worldwide and, as well as being recognised internationally as the leading cause of disability, it is a major contributor to the global burden of disease ^2^. Depression, in severe cases, can lead to suicide and almost 800,000 people worldwide each year die from suicide ^3^. The risk of suicide is almost 20 times higher in depressed patients than in the general population ^4^. Life expectancy is lowered by approximately 10 years in those with depression compared with the general population due to depression being a risk factor for many chronic cardiovascular and neurological conditions and also being associated with risky behaviours such as drug, alcohol and tobacco use ^4^.

Over the past 60 years, pharmacological treatment for depression has focused on drugs targeting monoamine neurotransmitters, including tricyclic antidepressants (TCAs), selective serotonin reuptake inhibitors (SSRIs) and serotonin-norepinephrine reuptake inhibitors (SNRIs) ^5^. These treatments, however, can take up to six weeks to have an effect. Additionally, multiple studies conducted in patients with depression indicate that less than 40% of patients treated with standard antidepressants achieved remission within 10–14 weeks, thereby highlighting the need for novel treatment approaches and faster-acting antidepressants ^6^.

One potential answer in addressing the need for more rapid and powerful antidepressants is the dissociative anaesthetic ketamine. Ketamine was approved by the Food and Drug Administration (FDA) in 1970 and since then has been used routinely as an anaesthetic ^7^. Ketamine is a racemic mixture that consists of two enantiomers, S- and R-ketamine. Ketamine acts as a non-competitive antagonist of the N-methyl-D-asparate (NMDA) receptor and thus targets the excitatory amino acid neurotransmitter glutamate ^8^. It is postulated that it is via this mechanism that its antidepressant properties are mediated, although this is not entirely clear and may involve its metabolites and downstream signalling events ^9^. There are other potential mechanisms that may contribute to ketamine’s antidepressant effects such as activation of the opioid system ^10^.

Ketamine has been shown to have rapid-acting but short-lived antidepressant effects ^11^. Since the initial reporting of ketamine’s antidepressant effect in 2000 by Berman *et al.* ^12^, most published trials using ketamine as an antidepressant have used similar low subanaesthetic doses (0.5mg/kg) and the same route of administration (i.e. a single slow intravenous infusion over 40 minutes). A single infusion of low dose subanaesthetic ketamine rapidly improves depressive symptoms with efficacy onset within one hour post infusion, peak effect sizes occur at 24 hours and last up to 8 days ^13^. Other doses of ketamine have been investigated to assess for superior antidepressant efficacy. It appears that doses less than 0.3mg/kg are ineffective while doses of 1mg/kg are intolerable due to dissociative side-effects, suggesting that 0.5mg/kg intravenous ketamine is an optimal dose with regards to therapeutic effect and tolerability ^14^. Other routes of ketamine administration have also been investigated including intramuscular, subcutaneous and oral; however, these have been found to be less effective than the intravenous route, possibly due to reduced bioavailability ^15, 16^. Very few trials have used intravenous ketamine as an adjunctive, or add-on, treatment for depression with most requiring a washout period of antidepressant prior to commencement of ketamine. Results from two randomised trials show that a single intravenous ketamine infusion, added to oral antidepressant therapy, has a more rapid antidepressant response than placebo (saline) ^17, 18^.

Sub-anaesthetic doses of ketamine are generally safe and well tolerated but can have short-lived physical, psychotomimetic, neurological and psychiatric side-effects ^19^. These adverse effects peak within the first two hours of the infusion and tend to resolve within 4–24 hours ^20^. Psychotomimetic side-effects are the most commonly reported with dissociation being the most prevalent. Psychiatric side effects, particularly anxiety, are also frequently reported and, like dissociation, can result in participants withdrawing from trials ^19^. Less frequently reported are cardiovascular side-effects, which include increase in blood pressure and heart rate due to ketamine’s activation of the sympathetic nervous system ^21^ while headaches and dizziness are among the most common neurological side-effects. Limited use of sub-anaesthetic doses of ketamine is safe with side-effects being transient; however chronic, mostly recreational, high-dose ketamine use can cause uropathy and dependency ^22^.

To date, there have been only three reported randomised controlled trials that have used repeated serial dosing of intravenous ketamine (once to thrice weekly for up to four weeks) to prolong its antidepressant effect. One trial (n=67) used twice and thrice weekly dosing of ketamine versus placebo (saline) for four weeks in medicated outpatients with treatment resistant depression, with a three-week follow-up period ^23^. Depression scores were significantly reduced and more sustained in both ketamine groups compared with the placebo group. Another randomised trial (n=26) administered six ketamine infusions over three weeks, versus placebo (saline), to medicated outpatients with depression and co-morbid suicidal ideation ^24^. Follow-up was continued for three months after the final infusion. Results showed that ketamine was not superior over placebo in reducing depression scores. A very recent clinical trial (n=54) compared a single ketamine infusion (preceded by five midazolam (active placebo) infusions) to six repeated ketamine infusions in medicated participants with diagnosed treatment resistant depression ^25^. Ketamine had a greater antidepressant effect over midazolam. However, there was no significant difference in the median time to relapse between the single and repeated ketamine infusions. Although numbers in this trial were small, it indicates that serial infusions may not be advantageous over a single infusion in prolonging ketamine’s antidepressant effect. The positive effects of serial ketamine infusions therefore currently remain uncertain.

Intranasal esketamine has had some promising results in terms of its rapid improvement of depressive symptoms and ease of administration ^26^. It has recently become available on the market in the US and EU for use as an adjunctive treatment in treatment resistant depression. Repeated adjunctive intranasal esketamine, administered twice-weekly, then tapering in frequency over a ten-week period, showed rapid antidepressant effects with a sustained response ^27^. Similarly, serial ketamine infusions, as an adjunctive treatment, have a rapid antidepressant effect, and this effect may be maintained for longer than a single infusion ^23^. Reporting of serial ketamine infusions as an adjunctive treatment to standard routine care is limited. 

We wished to investigate ketamine as an adjunctive treatment to improve recovery in patients hospitalised with depression. The aim of this study was to conduct a pragmatic randomised controlled patient- and rater-blinded pilot trial of a four-week course of once-weekly infusions of ketamine compared to midazolam as an adjunctive therapy for depression. All other treatments and therapies continued as usual to ensure a greater degree of generalisability. The main objective of this pilot trial was to assess trial procedures to inform a future definitive trial ^28^, including rates of recruitment, dropout and completion of follow-up assessments. Additionally, we sought to establish a 95% confidence interval for the differences between the ketamine and midazolam groups at the end of treatment time point. We hypothesised that the trial protocol would be feasible. 

## Methods

### Study design and participants

The Karma-Dep (Ketamine as an adjunctive therapy for major depression) trial was a randomised controlled pragmatic pilot trial of four once-weekly ketamine infusions (0.5 mg/kg over 40 minutes) compared to midazolam infusions (0.045 mg/kg over 40 minutes) as an adjunctive therapy in hospitalised depressed patients. Midazolam was used as an active placebo to help with blinding ^14^. Patients who were admitted to St Patrick’s University Hospital for treatment of a depressive episode between September 2017 and June 2018 and who met eligibility criteria for the trial were approached by the research team within 10 days of admission. Depressive episodes were diagnosed using the Diagnostic and Statistical Manual of Mental Disorders – fifth edition, DSM-5 ^29^, and confirmed using the Mini International Neuropsychiatric Interview (MINI; updated Version 7 for DSM-5) ^30^. After participants provided written and informed consent, they were invited to be randomised to a course of four once-weekly ketamine or midazolam (active comparator) infusions that began at the next clinic, which took place every Wednesday morning in St Patrick’s University Hospital. Treatment-as-usual, directed by the participants’ treating teams, continued throughout the entire trial. Psychotomimetic, physical, and cognitive outcomes were monitored before, during and after infusions. Participants were followed up for 12 weeks after the final infusion.

This clinical trial was approved by the Research Ethics Committees of both the Mater Misericordiae Hospital, Dublin, and St Patrick’s University Hospital, Dublin. This trial was also approved by the Health Products Regulatory Authority (HPRA) of Ireland, the relevant national body under the European framework for clinical trials (EudraCT 2016-004764-18 on 30/11/2016) and was registered with ClinicalTrials.gov (NCT03256162 on 21/08/2017).

Eligible participants were inpatient ≥18 years old, being treated for an acute depressive episode and met DSM-5 criteria for major depressive disorder (MDD) or bipolar affective disorder (current episode depression) and scored ≥21 on the 24-item Hamilton Rating Scale for Depression (HRSD-24). To be eligible for inclusion, subjects must have met each of the above criteria at screening and baseline assessments. Exclusion criteria were current involuntary admission, active suicidal intention, dementia, history of Axis 1 diagnosis other than a major depressive episode, ECT administered within the last two months, a Montreal Cognitive Assessment (MoCA) score <24, alcohol/substance dependence in the previous six months, pregnancy or inability to confirm use of adequate contraception during the trial, breastfeeding women and any medical condition rendering the patient unfit for ketamine/midazolam. Contraindications to ketamine, as per summary of product characteristics, included hypersensitivity to the active substance, uncontrolled hypertension, severe coronary or myocardial disease, cerebrovascular accident and cerebral trauma. Contraindications to midazolam included known hypersensitivity to benzodiazepines, severe respiratory failure or acute respiratory depression. All candidates meeting any of the exclusion criteria at screening/baseline were excluded from study participation.

### Randomisation and blinding

Block randomisation was performed by another researcher within St Patrick’s University Hospital and who was not otherwise associated with the Karma-Dep pilot trial. Randomisation was done in blocks of two and four. This randomisation list was stored in a locked cabinet to which only the anaesthetist (ES) had access. A protocol was in place to allow emergency unblinding.

Study treatment assignment was blinded for both the raters and the participants as well as the regular treating teams. To ensure patient safety during infusions and in the post-infusion period, the anaesthetist administering the ketamine/midazolam infusions was not blinded but was not involved in assessments or data analysis. Infusions were prepared by the anaesthetist in a location separate to the infusion area and labelled as “trial infusion” prior to transfer to the infusion area. Success of blinding for patients and raters was assessed after the first treatment.

### Assessments

The primary clinical outcome measure for this study was the change in the 24-item Hamilton Rating Scale for Depression (HRSD-24) ^31^. HRSD-24 scores were obtained 60 minutes (-60 mins) before the infusion began (0 mins) and at 120 and 240 minutes after the start of the infusion. Scores on items such as sleep and appetite were carried over from the -60 mins HRSD-24 as they were unable to change in such a short period of time. The first pre-infusion HRSD-24 scores served as the baseline scores while subsequent pre-infusion scores served as weekly HRSD-24 scores with the end of treatment score taken one week after the final infusion. Follow-up assessments using the HRSD-24 were completed at six and 12 weeks after the final infusion. HRSD-24 interrater reliability was assessed before the study began and six-months into the recruitment period with intraclass correlation coefficient scores of ≥0.95.

*Response* to treatment was defined as achieving a ≥60% decrease from baseline HRSD-24 and a score ≤16. *Remission* criteria were ≥60% decrease in HRSD-24 from baseline and a score ≤10 on two consecutive weekly ratings. Criteria for *relapse* were a ≥10 point increase in HRSD-24 compared to responders’ end of treatment score and a HRSD-24 score of ≥16. A readmission to hospital for psychiatric treatment or episode of self-harm was also considered a relapse of a depressive episode. The 16-item Quick Inventory of Depressive Symptoms, self-report version (QIDS-SR 16) was used at all the same time points as the HRSD-24 to measure subjective depression ratings ^32^.

Patient demographic information was collected pre-randomisation and included age, weight, education, marital status and socioeconomic group. Clinical variables included age of onset of depression, previous number of episodes, duration of index episode, prescribed psychotropic medications and length of hospital stay. Additional baseline assessments included the Maudsley Staging Method for Treatment Resistant Depression (MSMTRD), which is scored from 3 to 15 with higher numbers indicating a greater degree of treatment resistance ^33^. 

To capture any side-effects participants may have experienced during or after the infusion period, we used a number of instruments before (-60mins), during (+30 mins) and after (+60 mins) the infusions. The Clinician-Administered Dissociative States Scale (CADSS) ^34^ was used to capture dissociative effects and is scored out of a maximum of 92. The positive symptoms subscale of the Brief Psychiatric Rating Scale (BPRS) was used to measure psychotomimetic effects ^35^. This 4-item positive symptoms subscale measures suspiciousness, hallucinations, unusual thought content and conceptual disorganisation and is scored out of a maximum of 28. We used the Young Mania Rating Scale (YMRS; mood item) ^36^ to assess for mood elevation with a maximum score of 4.

Heart rate, blood pressure, pulse oximetry, and ECG were recorded to measure haemodynamic changes before and during infusions and for a further 200 minutes. These vital signs measures were taken at baseline and every 10 minutes during the infusion period. When the infusion ended, they were taken every 20 minutes for a further 80 minutes and then every 40 minutes until the end of the four-hour monitoring period. Infusions were discontinued by the anaesthetist if there were persisting haemodynamic changes (i.e. heart rate >110/minute or systolic/diastolic blood pressure >180/100 or >20% increase above pre-infusion BP for more than 15 minutes) that did not respond to beta-blocker therapy.

 The Patient-Rated Inventory of Side Effects (PRISE) was used to document other general adverse events by patients before (-60mins), during (+30 mins) and after (+60 mins) infusions ^37^.

Cognitive function was assessed using the Montreal Cognitive Assessment (MoCA) ^38^ at baseline, one day after the first and final infusions and at the final follow-up 12 weeks after the last infusion.

### Interventions

Ketamine (0.5 mg/kg) and midazolam (0.045 mg/kg) were made up as 50 ml colourless saline solutions and administered as slow infusions over 40 minutes using an infusion pump, as previously described ^39^. Like ketamine at 0.5 mg/kg, midazolam at 0.045 mg/kg has anaesthetic effects and causes some sedation with a similar time course. Participants were asked to fast for 8 hours prior to infusion clinics. All patients were monitored for heart rate, blood pressure, pulse oximetry and ECG before, during and after infusions. A researcher contacted each participant 24 hours after each infusion to check for additional potential adverse effects.

Patients were withdrawn from the trial if an infusion was discontinued for haemodynamic reasons or other serious medical contra-indications, e.g. over-sedation, hypoxia, intolerable adverse physical reactions; the patient developed mania or psychosis; the patient became severely depressed and/or suicidal. All infusions took place while participants were inpatients.

### Statistical analysis

Baseline clinical and demographic characteristics are presented using descriptive statistics. Variables were examined for normality using the Kolmogorov-Smirnov test. 95% confidence intervals for the differences between the groups at the end of treatment phase were calculated. Descriptive and comparative statistics were performed using IBM SPSS Statistics, version 24.0 (IBM Corp, Armonk, NY). We initially planned to recruit 40 participants (20 per group) to also allow for some biomarker research ^40^. However, recruitment ended earlier than expected when 25 participants had been enrolled in good time, demonstrating that a larger definitive trial was clearly feasible. A sample size of 12 participants per group is adequate for the purpose of a pilot trial ^41^. Data are presented as means (standard deviation (SD)) or medians (range).

## Results

### Participants

**Figure 1.**
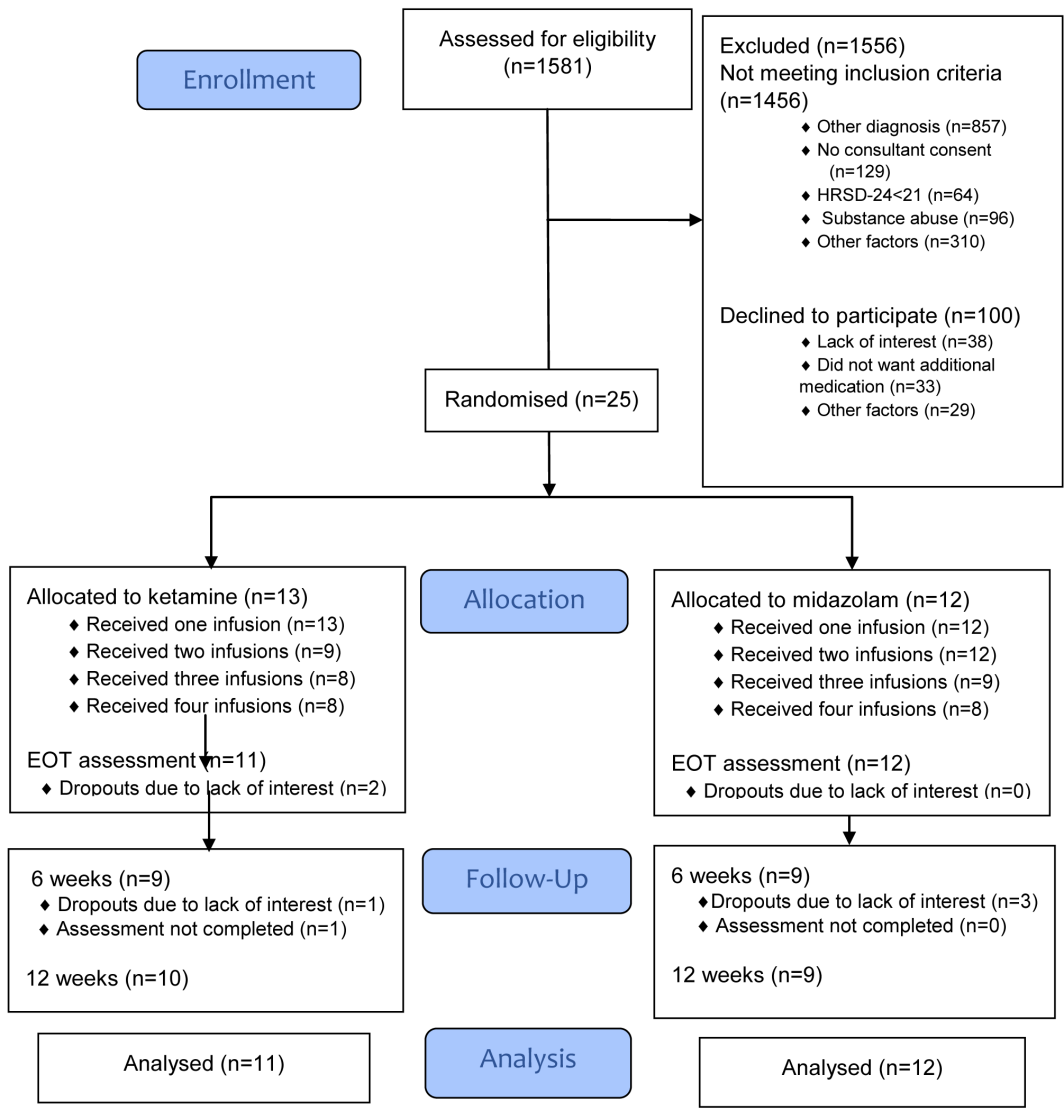
CONSORT flow diagram. EOT, end of treatment.

The CONSORT diagram in Figure 1 summarises reasons for ineligibility, non-recruitment and dropouts at each stage of the trial. Recruitment took place over a nine-month period between September 2017 and June 2018 and follow-up assessments were completed by September 2018. During this time, 1581 admissions to St Patrick’s University Hospital were assessed for eligibility. Of these, 125 (8%) were potentially eligible to participate in the trial and 25 (20%) of these agreed to participate. In total, 13 patients were randomly assigned to the ketamine arm and 12 to the midazolam arm. Demographic and baseline clinical characteristics of the sample are summarised in Table 1. The randomised groups were reasonably balanced with no major obvious differences between age, gender, baseline depression scores and level of treatment resistance.

**Table 1.** Baseline clinical and demographic characteristics. BMI indicates body mass index; HRSD-24, 24-item Hamilton Rating Scale for Depression; QIDS-SR-16, Quick Inventory of Depressive Symptoms, Self-Report, 16-item; MOCA, Montreal Cognitive Assessment; MSTRD, Maudsley Staging Method for Treatment Resistance in Depression; ECT, Electroconvulsive therapy; SSRI, Selective Serotonin Reuptake Inhibitors.

Characteristic	Total sample (n=25)		Ketamine (n=13)		Midazolam (n=12)	
	Mean	SD	Mean	SD	Mean	SD
**Age (years)**	50.5	12.6	48.9	13.1	52.3	12.5
**BMI**	28.7	5.5	28.2	7.1	29.3	3.1
**Education (years)**	15.9	3.4	17.1	3.9	14.7	2.2
**Baseline HRSD-24**	27.7	3.9	28.4	4.3	27.4	4.3
**Baseline QIDS-SR 16**	17.2	3.9	17.3	3.3	16.8	4.6
**Baseline MOCA**	26.9	1.9	26.8	2.0	27.0	1.9
**Treatment resistance (MSTRD)**	7.4	2.1	7.8	2.5	7.0	1.5
**Number of psychotropic medications**	3.2	1.5	3.6	1.5	2.8	1.4
	Median	Range	Median	Range	Median	Range
**Age of onset of depression**	21	12-49	21	12-43	22	12-49
**Previous number of episodes**	5	1-40	5	2-40	7	1-20
**Episode duration (in days)**	60	14-720	42	14-330	75	14-720
**Length of stay**	35	8-107	37	8-107	34.5	11-63
	N	%	N	%	N	%
**Female**	13	52.0	8	61.5	5	41.7
**Marital status:**						
** Married**	11	44.0	7	53.8	4	33.3
** Single**	8	32.0	2	15.4	6	50.0
** Widowed/divorced**	6	24.0	4	30.8	2	16.7
**Socioeconomic group:**						
** Professional**	5	20.0	4	30.8	1	8.3
** Managerial or technical**	11	44.0	5	38.5	6	50.0
** Skilled occupation**	4	16.0	2	15.4	2	16.7
** Partly skilled occupation**	3	12.0	2	15.4	1	8.3
** Unskilled occupations**	2	8.0	0	0	2	16.7
**Previous ECT, yes**	4	16.0	2	15.4	2	16.7
**Psychotropic medications**						
** SSRIs**	9	36.0	5	38.5	4	33.3
** Non-SSRIs**	20	80.0	11	84.6	9	75.0
** Mood stabilisers**	10	40.0	6	46.2	4	33.3
** Antipsychotics**	13	52.0	10	76.9	3	25.0
** Benzodiazepines**	12	48.0	5	38.5	7	58.3
** Hypnotics**	9	36.0	4	30.8	5	41.7

A total of 16 participants (64%) completed all four infusions, eight in each group. In the ketamine group, four (30.8%) participants discontinued after one infusion. Of these, two dropped out due to dissociative side-effects, one chose to do a therapeutic programme and could no longer attend the infusion clinics and one had improved sufficiently to be discharged from hospital. One participant (7.8%) discontinued after two infusions as they were prescribed electroconvulsive therapy (ECT). In the midazolam group, three participants (25%) discontinued after the second infusion. One was due to side-effects (nightmares), one was referred for ECT and one had improved sufficiently to be discharged from hospital. One (8.3%) discontinued after the third infusion as they left hospital against medical advice. End of treatment assessments were obtained for 11/13 (85%) of patients in the ketamine group and 12/12 (100%) in the midazolam group. Retention rates of participants who agreed to remain in the trial for follow-up purposes were as follows: 18/25 (72%) at six weeks and 19/25 (76%) after 12 weeks. Retention rates in the ketamine group were 9/13 (69%) at six weeks and 10/13 (77%) at 12 weeks. In the midazolam group rates were 9/12 (75%) at week six and 9/12 (75%) at week 12. 

When asked about treatment allocation after the first infusion session, 10/13 (76.9%) in the ketamine group and 7/12 (58.3%) in the midazolam group correctly guessed their treatment. For raters, 10/13 (76.9%) guesses for the ketamine group and 10/12 (83.3%) for the midazolam group were correct. 

### Depression outcomes

**Figure 2.**
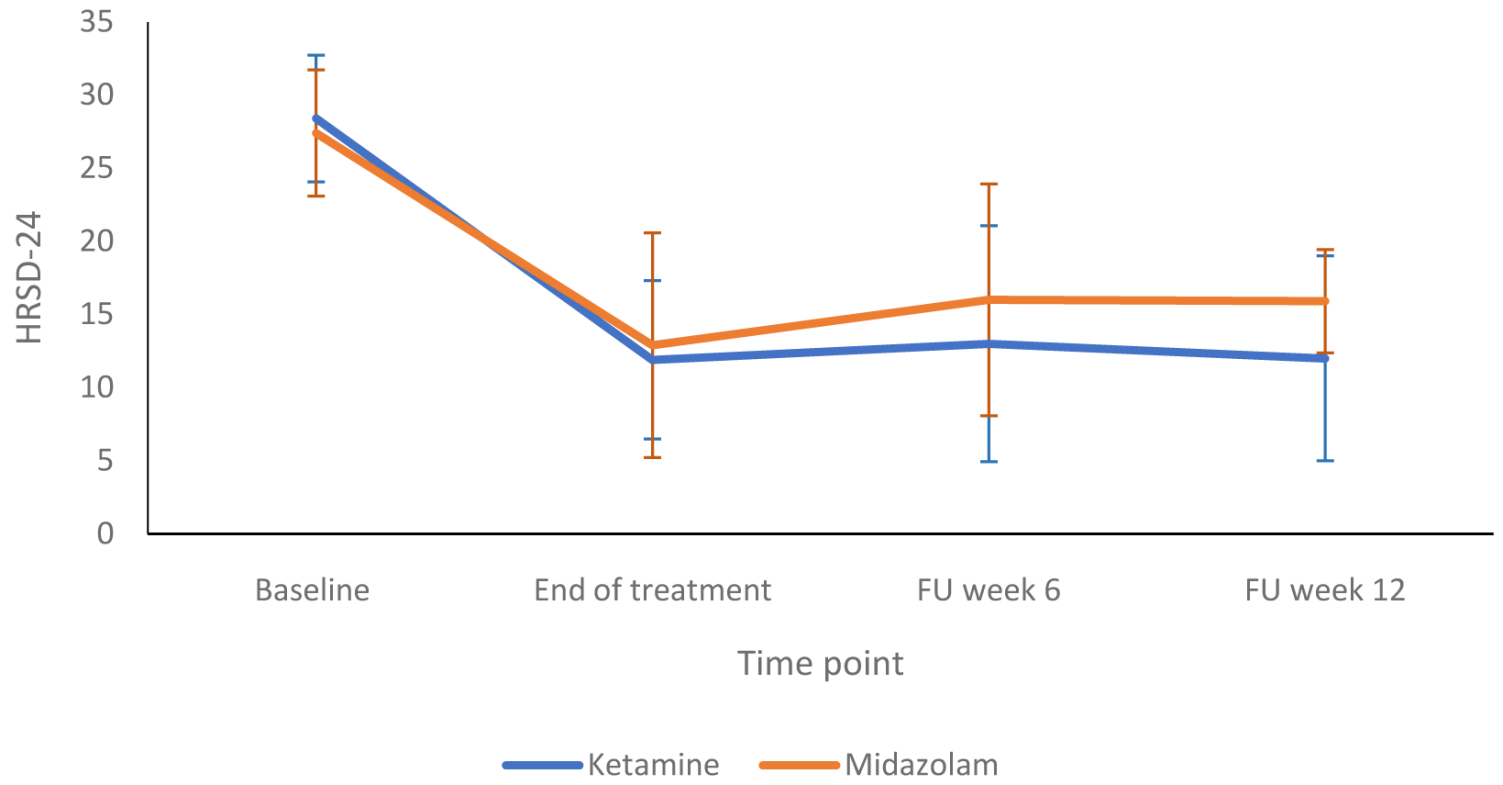
Depression rating scores at end of treatment and follow up timepoints: 24-item Hamilton Rating Scale for Depression (HRSD-24) scores are presented as mean (SD) values. FU, follow-up. Number of participants at each stage: Baseline - Ketamine n=13, Midazolam n=12; End of treatment - Ketamine n=11, Midazolam n=12; Follow-up at 6 weeks - Ketamine n=9, Midazolam n=9; Follow-up at 12 weeks - Ketamine n=10, Midazolam n=9.

**Figure 3.**
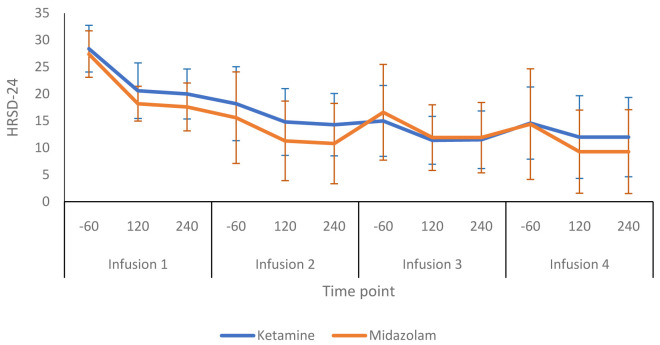
Depression rating scores throughout the infusion sessions: 24-item Hamilton Rating Scale for Depression (HRSD-24) scores are presented as mean (SD) values. -60, 60 minutes before infusion begins; 120, 120 minutes post start of infusion; 240, 240 minutes post start of infusion. Number of participants at each infusion: Infusion 1 - Ketamine n=13, Midazolam n=12; Infusion 2 - Ketamine n=9, Midazolam n=12; Infusion 3 - Ketamine n=8, Midazolam n=9; Infusion 4 - Ketamine n=8, Midazolam n=8.

**Figure 4.**
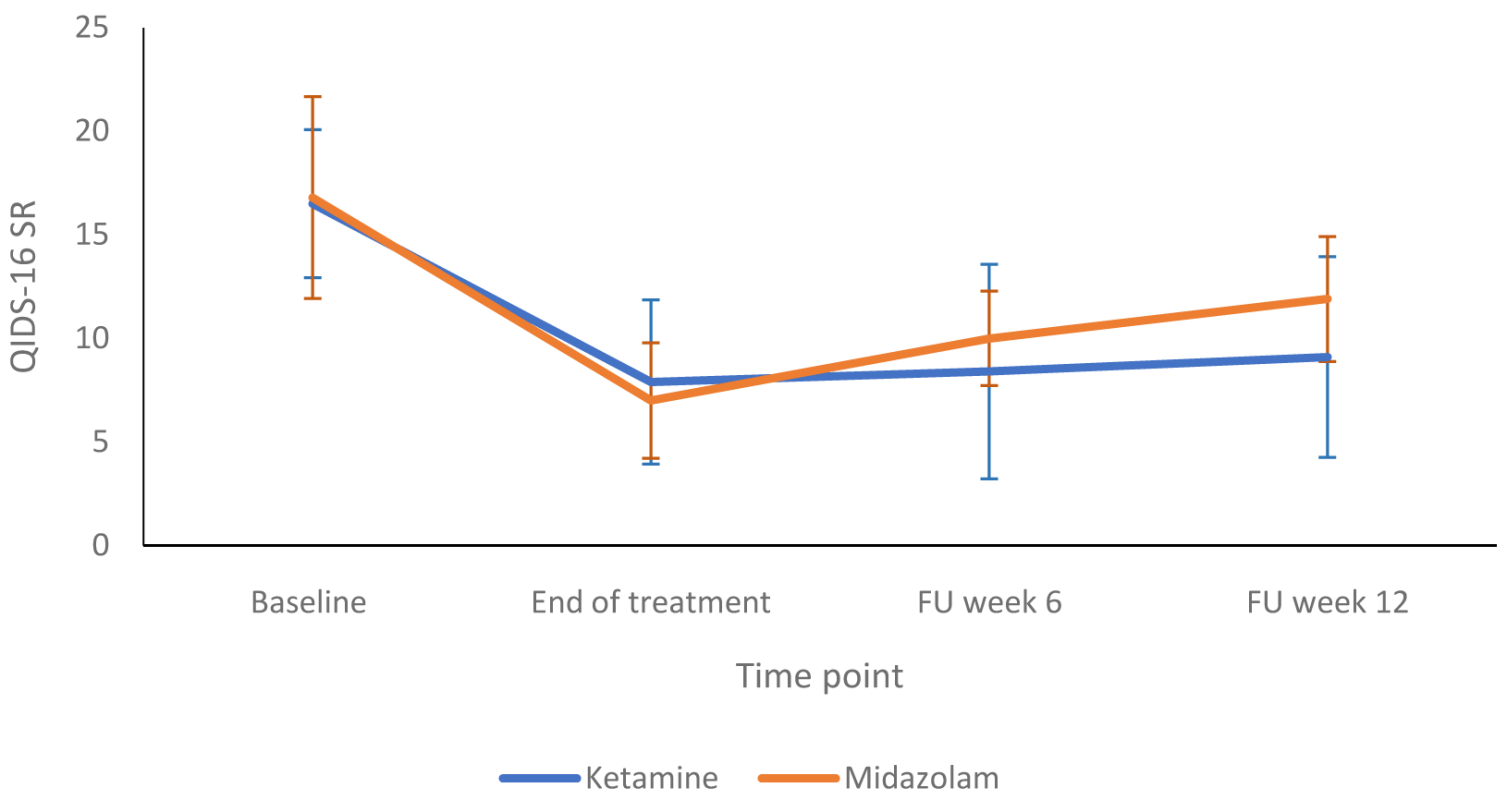
Patient-rated depression scores at end of treatment and follow up timepoints: 16-item Quick Inventory of Depressive Symptoms, self-report version, (QIDS-SR 16) scores are presented as mean (SD) values. FU, follow-up. Number of participants at each stage: Baseline - Ketamine n=13, Midazolam n=12; End of treatment - Ketamine n=11, Midazolam n=12; Follow-up at 6 weeks - Ketamine n=9, Midazolam n=9; Follow-up at 12 weeks - Ketamine n=10, Midazolam n=9.

**Figure 5.**
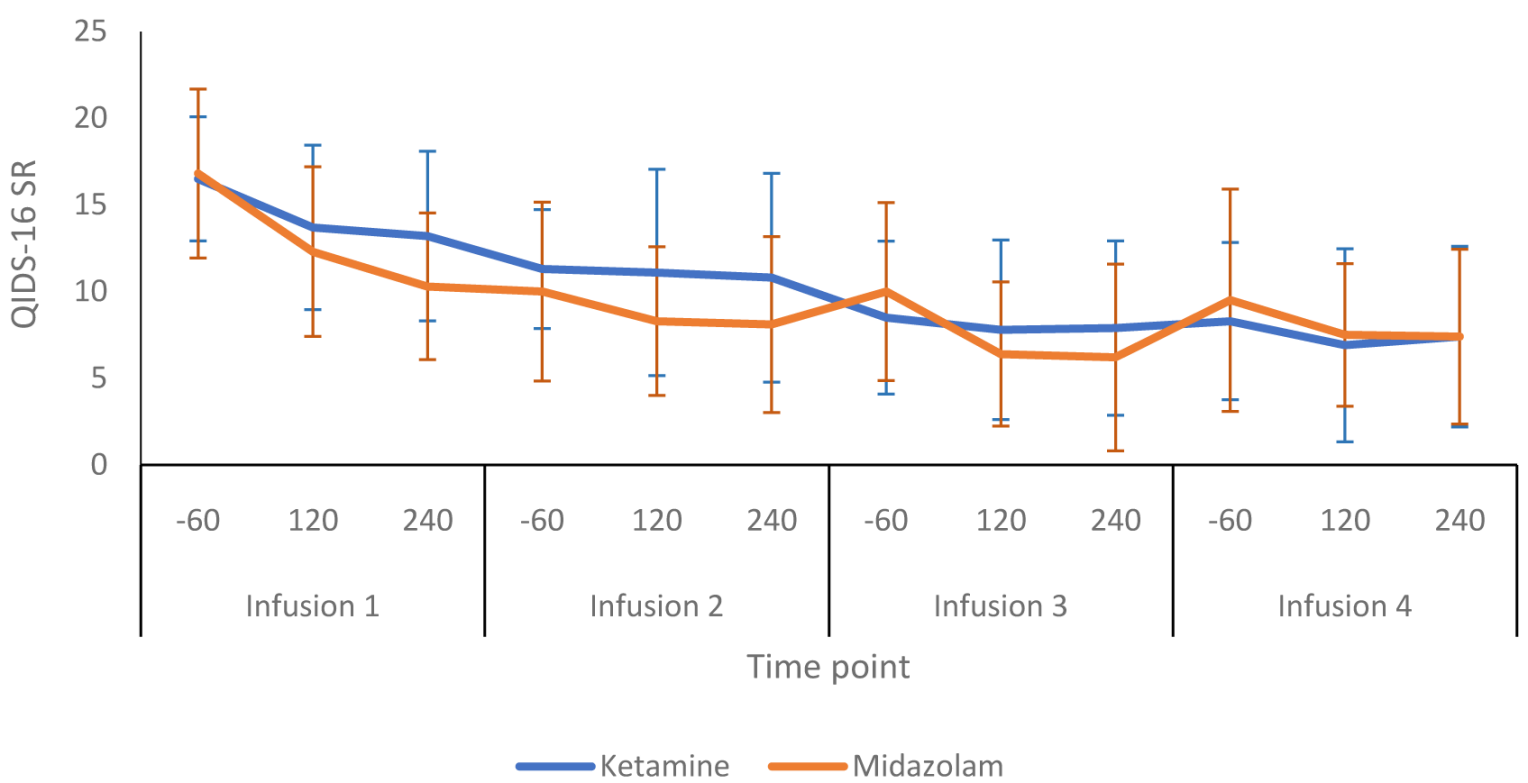
Patient-rated depression scores throughout the infusion sessions: 16-item Quick Inventory of Depressive Symptoms, self-report version, (QIDS-SR 16) scores are presented as mean (SD) values. -60, 60 minutes before infusion begins; 120, 120 minutes post start of infusion; 240, 240 minutes post start of infusion. Number of participants at each infusion: Infusion 1 - Ketamine n=13, Midazolam n=12; Infusion 2 - Ketamine n=9, Midazolam n=12; Infusion 3 - Ketamine n=8, Midazolam n=9; Infusion 4 - Ketamine n=8, Midazolam n=8.

Figure 2 compares the HRSD-24 scores in the ketamine and midazolam groups at baseline, end of treatment and follow up at weeks six and 12. Both groups followed a similar pattern, with depression scores lower at end of treatment than at baseline. Figure 3 shows a more detailed breakdown of the HRSD-24 scores for both groups at -60, +120 and +240 minutes during infusion sessions 1 to 4. The mean difference in HDRS-24 scores between the ketamine and midazolam groups, at the end of the treatment phase was -2.6 (95% CI -8.26 to 3.03) points. The QIDS-16 SR scores followed a similar pattern to the HRSD-24 scores, as shown in Figure 4 and Figure 5. Data relating to the very first infusion sessions have been previously reported on in a study examining peripheral blood molecular changes related to ketamine ^40^.

The rates of responders and remitters in both groups are shown in Table 2. There were no participants, in either group, who met response criteria during the first infusion session at either +120 or +240 minutes post start of infusion. At the end of treatment assessment, the proportion of responders in the ketamine and midazolam groups were 6/11 (55%) and 6/12 (50%) while remission rates were 2/11 (18%) and 4/12 (33%), respectively. By the 12-week follow-up, the proportion of responders in the ketamine and midazolam groups were 7/10 (70%) and 4/9 (44%) and proportion of remitters were 2/10 (20%) and 2/9 (22%) respectively.

**Table 2.** Rates of responders and remitters during the treatment phase and follow-up timepoints. Inf, Infusion; +120, 120 minutes post commencement of infusion: +240, 240 minutes post commencement of infusion; EoT, End of treatment; FU, Follow-up.

	Response (%)		Remission (%)	
	Ketamine	Midazolam	Ketamine	Midazolam
**Inf 1: +120 mins**	0/13 (0%)	0/12 (0%)	0/13 (0%)	0/12 (0%)
**Inf 1: +240 mins**	0/13 (0%)	0/12 (0%)	0/13 (0%)	0/12 (0%)
**Inf 2: baseline**	2/13 (15%)	4/12 (33%)	0/13 (0%)	0/12 (0%)
**Inf 3: baseline**	2/8 (25%)	3/9 (33%)	1/9 (11%)	3/9 (33%)
**Inf 4: baseline**	2/8 (25%)	3/8 (38%)	2/8 (25%)	3/8 (38%)
**EoT assessment**	6/11 (55%)	6/12 (50%)	2/11 (18%)	4/12 (33%)
**FU week 6**	5/9 (56%)	2/9 (22%)	3/9 (33%)	2/9 (22%)
**FU week 12**	7/10 (70%)	4/9 (44%)	2/10 (20%)	2/9 (22%)

### Safety and tolerability

**Figure 6.**
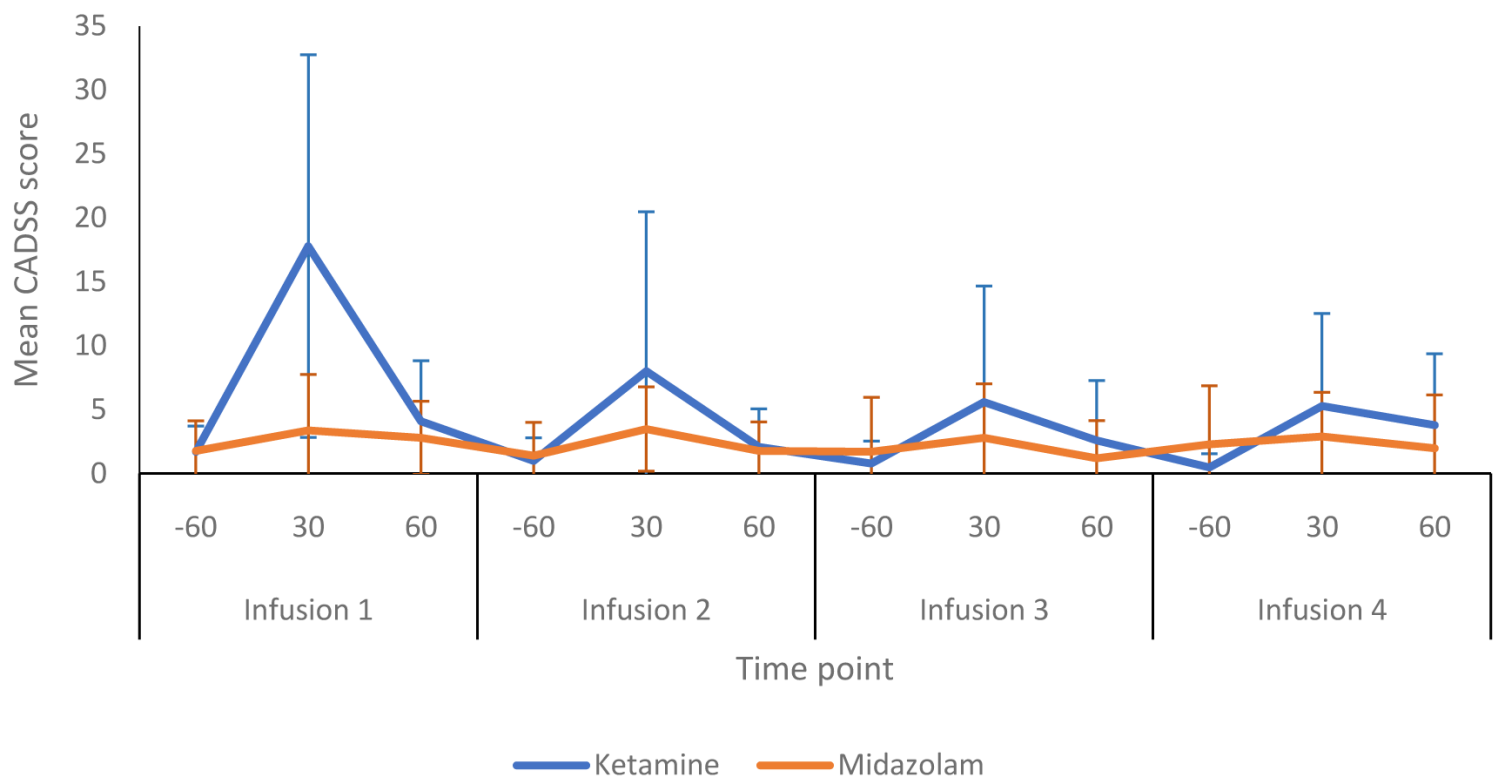
Dissociative symptoms during infusion sessions: The graph shows the total mean (SD) scores for the Clinician-Administered Dissociative States Scale (CADSS) during allocated infusion sessions. -60, 60 minutes prior to commencement of infusion; 30, 30 minutes post start of infusion: 60, 60 minutes post start of infusion. Number of participants at each infusion: Infusion 1 - Ketamine n=13, Midazolam n=12; Infusion 2 - Ketamine n=9, Midazolam n=12; Infusion 3 - Ketamine n=8, Midazolam n=9; Infusion 4 - Ketamine n=8, Midazolam n=8.

Several instruments were used before, during and after infusion sessions to assess for psychotomimetic side-effects. Figure 6 summarises the CADSS dissociative symptom scores obtained from the ketamine and midazolam groups at various time points during infusion sessions. Participants receiving ketamine tended to score higher than those in the midazolam group. During the first infusion, the mean CADSS score (at 30 minutes) was 17.8 (15.0) in the ketamine group and 3.4 (4.4) in the midazolam group. The peak CADSS scores within the ketamine group declined with each subsequent infusion session and returned towards pre-infusion levels at the +60 minute time point (i.e. 20 minutes after infusions finished) for each of the four infusions sessions ( Table 3). Psychosis (BPRS) and mania (YMRS) scores are shown in Table 3. No participants in either group experienced psychosis or elevated mood during any of the infusion sessions.

**Table 3.** Emergent dissociation, psychosis and mania during infusion sessions. CADSS, Clinician-Administered Dissociative States Scale presented as mean (SD) values; BPRS, the positive symptoms subscale of the Brief Psychiatric Rating Scale presented as median (range) values; YMRS, Mood item of the Young Mania Rating Scale; -60, 60 minutes prior to start of infusion; +30, 30 minutes post start of infusion; +60, 60 minutes post start of infusion; Number of participants at each infusion: Infusion 1 - Ketamine n=13, Midazolam n=12; Infusion 2 - Ketamine n=9, Midazolam n=12; Infusion 3 - Ketamine n=8, Midazolam n=9; Infusion 4 - Ketamine n=8, Midazolam n=8.

	Time point	CADSS		BPRS		YMRS	
		Ketamine	Midazolam	Ketamine	Midazolam	Ketamine	Midazolam
**Infusion 1**	-60	1.7 (2.0)	1.8 (2.3)	4 (4-4)	4 (4-7)	0	0
	+30	17.8 (15.0)	3.4 (4.4)	4 (4-6)	4 (4-6)	0	0
	+60	4.1(4.7)	2.8 (2.9)	4 (4-5)	4 (4-5)	0	0
**Infusion 2**	-60	1.0 (1.8)	1.4 (2.6)	4 (4-5)	4 (4-5)	0	0
	+30	8.0 (12.5)	3.5(3.3)	4 (4-5)	4 (4-5)	0	0
	+60	2.1 (3.0)	1.8 (2.2)	4 (4-5)	4 (4-4)	0	0
**Infusion 3**	-60	0.8 (1.8)	1.7 (4.3)	4 (4-5)	4 (4-6)	0	0
	+30	5.6 (9.1)	2.8 (4.2)	4 (4-5)	4 (4-5)	0	0
	+60	2.6 (4.7)	1.2 (2.9)	4 (4-5)	4 (4-5)	0	0
**Infusion 4**	-60	0.5 (1.1)	2.3 (4.6)	4 (4-4)	4 (4-4)	0	0
	+30	5.3 (7.2)	2.9 (3.5)	4 (4-5)	4 (4-5)	0	0
	+60	3.8 (5.6)	2.0 (4.2)	4 (4-4)	4 (4-5)	0	0

Table 4 shows the numbers of participants who experienced physical symptoms during each of the four infusion sessions as recorded by the PRISE. The most common new-onset side-effects that occurred during the first infusion session in the ketamine group were anxiety (31%), dizziness (31%), restlessness (23%), fatigue (23%), poor co-ordination (23%) and dry mouth (23%). These symptoms became less prevalent in subsequent infusion sessions. One participant reported new onset palpitations in the second ketamine infusion; however, this episode was short lived, was not distressing and was not described as a side-effect in subsequent infusions. There were no other new onset side-effects reported during subsequent ketamine infusions. The PRISE records whether the participants found the symptoms to be ‘tolerable’ or ‘distressing’. Two of the participants in the ketamine group reported anxiety as a new symptom in the first infusion session and found it to be distressing. The same two participants also experienced nausea and vomiting and reported this as distressing. Both participants dropped out of the trial after the first infusion session due to poor tolerability but agreed to be followed-up. All of the other symptoms reported in the ketamine group were considered to be tolerable.

**Table 4.** Number (%) of participants experiencing acute physical side-effects, as recorded by the PRISE, during each infusion session. K, ketamine; M, midazolam; Base, 60 minutes prior to each infusion; +30, 30 minutes post start of infusion; +60, 60 minutes post start of infusion; Number of participants at each infusion: Infusion 1 - Ketamine n=13, Midazolam n=12; Infusion 2 - Ketamine n=9, Midazolam n=12; Infusion 3 - Ketamine n=8, Midazolam n=9; Infusion 4 - Ketamine n=8, Midazolam n=8.

Symptom	Infusion 1						Infusion 2						Infusion 3						Infusion 4					
	K	M	K	M	K	M	K	M	K	M	K	M	K	M	K	M	K	M	K	M	K	M	K	M
	Base		+30		+60		Base		+30		+60		Base		+30		+60		Base		+30		+60	
Diarrhoea	0	0	0	0	0	0	0	0	0	0	0	0	0	0	0	0	0	0	0	0	0	0	0	0
Constipation	0	0	0	0	0	0	0	0	0	0	0	0	0	0	0	0	0	0	0	0	0	0	0	0
Dry mouth	1 (8)	1 (8)	4 (31)	2 (17)	1 (8)	1 (8)	1 (11)	0	2 (22)	0	1 (11)	0	0	0	1 (13)	0	0	0	0	0	1 (13)	1 (13)	0	0
Nausea / vomiting	0	0	2 (15)	0	0	0	0	0	0	0	0	0	0	0	0	0	0	0	0	0	0	0	0	0
Palpitation	0	0	0	0	0	0	0	0	1 (11)	0	0	0	0	0	0	0	0	0	0	0	0	0	0	0
Dizziness on standing	0	0	0	0	0	0	0	0	0	1 (8)	0	0	0	0	0	0	0	0	0	0	0	0	0	0
Chest pain	0	0	0	0	0	0	0	0	0	0	0	0	0	0	0	0	0	0	0	0	0	0	0	0
Rash	0	0	0	0	0	0	0	0	0	0	0	0	0	0	0	0	0	0	0	0	0	0	0	0
Increased perspiration	0	0	1 (8)	0	0	0	0	0	0	0	0	0	0	0	0	0	0	0	0	0	0	0	0	0
Itching	0	0	0	0	0	0	0	0	0	0	0	0	0	0	0	0	0	0	0	0	0	0	0	0
Dry skin	0	0	0	0	0	0	0	0	0	0	0	0	0	0	0	0	0	0	0	0	0	0	0	0
Headache	0	0	1 (8)	0	0	0	0	1 (8)	1 (11)	3 (25)	0	2 (17)	0	0	0	1 (11)	0	0	0	0	0	0	0	0
Tremors	0	0	1 (8)	0	0	0	0	0	0	0	0	0	0	0	0	0	0	0	0	0	0	0	0	0
Poor coordination	0	0	3 (23)	0	0	0	0	0	1 (11)	0	0	0	0	0	1 (13)	0	0	0	0	0	1 (13)	0	0	0
Dizziness	0	0	4 (31)	0	0	0	0	0	1 (11)	0	0	0	0	0	1 (13)	0	0	0	0	0	2 (25)	0	0	0
Blurred vision	0	0	2 (15)	1 (8)	0	0	0	0	1 (11)	1 (8)	0	0	0	0	1 (13)	0	0	0	0	0	0	0	0	0
Ringing in ears	0	0	1 (8)	0	0	0	0	0	0	0	0	0	0	0	0	0	0	0	0	0	0	0	0	0
Difficulty urinating	0	0	0	0	0	0	0	0	0	0	0	0	0	0	0	0	0	0	0	0	0	0	0	0
Painful urination	0	0	0	0	0	0	0	0	0	0	0	0	0	0	0	0	0	0	0	0	0	0	0	0
Frequent urination	0	0	0	0	0	0	0	0	0	0	0	0	0	0	0	0	0	0	0	0	0	0	0	0
Menstrual irregularity	0	0	0	0	0	0	0	0	0	0	0	0	0	0	0	0	0	0	0	0	0	0	0	0
Difficulty sleeping	0	0	0	0	0	0	0	0	0	0	0	0	0	0	0	0	0	0	0	0	0	0	0	0
Sleeping too much	1 (8)	1 (8)	1 (8)	4 (33)	1 (8)	1 (8)	0	0	1 (11)	1 (8)	0	0	0	0	2 (25)	1 (11)	0	0	0	0	0	1 (13)	0	0
Loss of sexual desire	0	0	0	0	0	0	0	0	0	0	0	0	0	0	0	0	0	0	0	0	0	0	0	0
Trouble achieving orgasm	0	0	0	0	0	0	0	0	0	0	0	0	0	0	0	0	0	0	0	0	0	0	0	0
Trouble with erections	0	0	0	0	0	0	0	0	0	0	0	0	0	0	0	0	0	0	0	0	0	0	0	0
Anxiety	2 (15)	2 (17)	6 (46)	3 (25)	2 (17)	1 (8)	1 (11)	0	3 (33)	0	1 (11)	0	0	0	0	0	0	0	0	0	2 (25)	0	1 (13)	0
Poor concentration	0	0	1 (8)	1 (8)	0	0	0	0	0	0	0	0	0	0	0	2 (22)	0	0	0	0	0	1 (13)	0	0
General malaise	0	0	0	0	0	0	0	0	0	0	0	0	0	0	0	0	0	0	0	0	0	0	0	0
Restlessness	0	0	3 (23)	0	0	0	0	0	0	0	0	0	0	0	1 (13)	0	0	0	0	0	2 (25)	0	0	0
Fatigue	1 (8)	1 (8)	4 (31)	5 (42)	1 (8)	1 (8)	0	0	2 (22)	3 (25)	0	1 (8)	0	1 (11)	1 (13)	5 (56)	0	2 (22)	1 (13)	0	1 (13)	3 (38)	1 (13)	0
Decreased energy	3 (23)	3 (25)	4 (31)	4 (33)	2 (17)	3 (25)	0	1 (8)	1 (11)	3 (25)	0	1 (8)	0	0	0	1 (11)	0	0	0	0	0	0	0	0

The most common side-effects that were reported during the first infusion session in the midazolam group were sleeping too much (33%) and fatigue (42%). Fatigue continued to be reported in the midazolam group in subsequent sessions with 38% reporting it in the fourth infusion. One participant reported new onset dizziness on standing during the second midazolam infusion; however, this was not reported in subsequent infusions. There were no participants who described any physical symptom in the midazolam group as ‘distressing’. There were no obvious differences in terms of new onset physical symptoms between the two groups.

Of the 32 physical symptoms that were specifically inquired about, there were no new onset of symptoms for 15 of these in either group during any of the four infusions. There were no participants who experienced new onset urinary symptoms in either group.

Vital signs were closely monitored and recorded throughout each infusion session. Table 5 shows the mean scores for systolic blood pressure (SBP), diastolic blood pressure, heart rate and oxygen saturation for baseline and 10, 20, 30, 40 and 60 minutes post start of infusion for each of the four infusion sessions in both groups. Mean changes in SBP from baseline to 40 minutes into the infusion were calculated for both groups during the first infusion session. In the ketamine group, the mean change in SBP between these two time points was 16.6 (11.5) mm Hg. In the midazolam group, the mean change was -13.3 (17.1) mm Hg. SBP tended to increase at 40 minutes in the ketamine group while SBP decreased at 40 minutes in the midazolam group. Nine participants in total (36%) had an increase in SBP of ≥20% from baseline during the first infusion session. Eight of these received ketamine and one received midazolam. Three participants (14%) had an increase in SBP of ≥20% from baseline during the second infusion – all three were in the ketamine group. Two participants (12%) had ≥20% increase in SBP from baseline during the third infusion session – one from each group, and, two participants (13%) had this increase in SBP in the fourth infusion session and both were in the midazolam group. Blood pressures were measured again 10 minutes after these elevated readings, and all elevated blood pressures had returned to within 20% of their baseline measures at this time. No pharmacological intervention was required for any of the increases in BP. There were no participants with a heart rate above 110 per minute during the infusion phase or up to 200 minutes after the infusion. Stable oxygen saturation and electrocardiogram rhythm were observed in all participants during the monitoring period before, during and after the infusions for up to 200 mins. There were no serious adverse events or reactions throughout the trial.

**Table 5.** Mean (SD) blood pressure, heart rate and oxygen saturation values for each infusion session. Ket, ketamine; Mid, midazolam; BP, blood pressure (mm Hg); HR, heart rate per minute; S02, oxygen saturation; Inf, infusion; 10 min, 10 minutes post start of infusion; 20 min, 20 minutes post start of infusion; 30 min, 30 minutes post start of infusion; 40 min, 40 minutes post start of infusion; 60 min, 60 minutes post start of infusion.

Timepoint		Systolic BP		Diastolic BP		HR		S02	
		Ket	Mid	Ket	Mid	Ket	Mid	Ket	Mid
**Inf 1**	**Baseline**	120.5 (17.6)	130.7 (14.9)	76.5 (10.6)	79.9 (12.8)	73.9 (12.4)	70.9 (12.6)	96.3 (2.0)	95.8 (2.5)
	**10 min**	127.5 (23.7)	121.8 (17.8)	79.5 (14.1)	75.2 (13.9)	74.5 (11.7)	69.3 (10.7)	97.0 (1.8)	95.3 (2.4)
	**20 min**	135.2 (24.1)	121.7 (21.3)	82.7 (13.6)	75.8 (15.2)	78.5 (11.8)	70.0 (12.6)	97.3 (2.1)	95.2 (2.6)
	**30 min**	137.8 (22.0)	117.3 (19.0)	85.2 (14.7)	75.1 (15.0)	78.3 (11.2)	69.5 (12.9)	96.9 (1.9)	94.9 (2.6)
	**40 min**	137.8 (25.2)	117.3 (19.0)	84.2 (12.4)	72.0 (16.8)	80.2 (15.1)	72.4 (15.0)	96.9 (2.6)	95.3 (2.6)
	**60 min**	131.1 (20.8)	124.2 (19.9)	76.3 (12.8)	73.7 (13.9)	75.2 (10.1)	72.4 (11.1)	96.6 (1.9)	95.5 (4.0)
**Inf 2**	**Baseline**	119.8 (16.6)	128.1 (16.2)	66.1 (10.2)	78.9 (13.6)	71.2 (22.4)	69.2 (68.3)	96.3 (2.0)	96.6 (2.0)
	**10 min**	124.9 (20.6)	118.4 (15.6)	68.0 (8.6)	73.5 (11.8)	74.9 (12.3)	67.1 (13.1)	95.9 (2.5)	95.6 (1.8)
	**20 min**	127.6 (18.0)	118.8 (15.7)	71.9 (8.5)	73.2 (11.4)	75.4 (10.1)	68.5 (12.0)	96.7 (1.9)	94.3 (2.2)
	**30 min**	128.4 (18.0)	113.0 (21.7)	73.4 (9.3)	68.9 (13.2)	78.6 (12.2)	68.7 (12.1)	96.4 (2.0)	94.8 (2.6)
	**40 min**	131.3 (18.0)	118.4 (14.5)	74.5 (9.8)	73.3 (6.6)	77.8 (10.4)	68.3 (11.5)	96.8 (1.7)	94.8 (2.9)
	**60 min**	124.6 (19.4)	118.8 (14.5)	67.9 (10.0)	75.8 (12.1)	77.9 (12.7)	68.7 (11.5)	95.4 (2.7)	95.6 (2.2)
**Inf 3**	**Baseline**	122.3 (20.0)	120.4 (16.6)	73.7 (8.1)	73.2 (13.7)	76.8 (13.0)	67.1 (13.3)	96.1 (2.7)	97.1 (1.5)
	**10 min**	124.0 (23.3)	117.1 (19.7)	73.9 (7.3)	73.3 (13.9)	73.8 (11.6)	65.0 (13.0)	95.5 (3.2)	94.8 (2.7)
	**20 min**	123.0 (21.2)	116.2 (20.5)	75.3 (7.4)	69.2 (15.2)	74.9 (11.2)	65.4 (12.2)	95.9 (3.1)	95.3 (2.7)
	**30 min**	125.5 (22.9)	117.7 (19.4)	78.0 (11.0)	70.0 (9.1)	77.1 (11.5)	64.3 (12.9)	95.8 (2.8)	93.9 (1.8)
	**40 min**	126.0 (23.6)	111.2 (17.1)	77.5 (8.6)	70.1 (12.7)	76.8 (11.5)	65.6 (13.0)	95.9 (2.5)	94.8 (2.7)
	**60 min**	125.9 (18.8)	117.0 (18.5)	72.7 (10.7)	73.1 (10.2)	73.5 (11.6)	65.8 (11.1)	96.6 (2.1)	95.1 (2.9)
**Inf 4**	**Baseline**	122.6 (17.6)	121.6 (22.2)	71.4 (10.3)	73.6 (15.7)	78.1 (9.6)	66.0 (12.6)	96.3 (1.4)	96.1 (1.6)
	**10 min**	122.6 (20.3)	115.5 (19.3)	72.4 (6.3)	71.0 (12.0)	77.5 (9.7)	62.8 (9.0)	95.9 (1.2)	94.8 (2.2)
	**20 min**	125.1 (23.3)	113.1 (18.6)	72.4 (4.8)	70.9 (13.3)	78.1 (9.4)	63.6 (9.8)	95.9 (1.9)	95.0 (0.9)
	**30 min**	128.1 (20.6)	115.4 (23.1)	73.9 (6.7)	68.3 (10.3)	79.1 (10.8)	64.8 (9.6)	95.5 (2.1)	94.6 (1.8)
	**40 min**	128.0 (18.8)	111.4 (18.9)	76.5 (5.8)	67.1 (11.2)	81.4 (10.3)	63.4 (10.5)	95.8 (2.2)	94.1 (1.9)
	**60 min**	125.1 (17.4)	116.1 (22.9)	74.2 (6.8)	72.1 (14.3)	81.4 (10.2)	65.5 (12.0)	95.8 (2.3)	94.0 (2.9)

The MoCA was used to assess cognitive function at four time points during the trial ( Table 6). There were no obvious differences between the scores of the two groups at the four time points and scores did not appear to differ from baseline.

**Table 6.** MoCA scores during treatment and at the 12 week follow up timepoint. Montreal Cognitive Assessment (MoCA) scores are presented as mean (SD) values; Number of participants at each timepoint: Baseline - Ketamine n=13, Midazolam n=12; 1 day post infusion 1 - Ketamine n=13, Midazolam n=12; 1 day post final infusion - Ketamine n=8, Midazolam n=8; 12 weeks follow-up - Ketamine n=8, Midazolam n=7.

Time point	Ketamine	Midazolam
**Baseline**	26.8 (2.0)	27.0 (1.9)
**1 day post infusion 1**	27.4 (1.6)	27.8 (1.6)
**1 day post final infusion**	27.6 (1.4)	27.8 (2.2)
**12 week follow up**	26.0 (3.0)	28.7 (1.4)

## Discussion

This is the first pragmatic trial of adjunctive serial ketamine infusions for hospitalised depression, an important possible use of ketamine. The aim of this pilot trial was to assess the feasibility of this study to inform a future definitive trial. Recruitment was successful and 20% of patients who were eligible for this study consented to take part. Adherence to interventions was also satisfactory with 8/13 participants completing all four ketamine infusions and 8/12 completing all four midazolam infusions. Overall, 16/25 (64%) of participants completed the intended four once-weekly infusions. Reasons for discontinuing included side-effects (3/9), commencing ECT (2/9), opting for doing an educational programme that coincided with clinic time (1/9) and being discharged from hospital before all four infusions were complete (3/9). Follow-up assessment rates were also adequate with 72% of participants completing the six-week and 76% completing the 12-week follow up.

In general, ketamine and midazolam infusions were safe and well tolerated in our small patient sample. Dissociative side-effects were more prevalent in the ketamine group in line with results from previous trials ^19^. With each subsequent infusion, dissociative symptoms in the ketamine group diminished. Some trials have experimented with various doses of ketamine (lower than 0.5mg/kg) and have shown that some participants respond to ketamine at doses as low as 0.1mg/kg ^16^. Dose titration of ketamine may be an alternative way of minimising side-effects whilst optimising efficiency and therefore reducing dropouts as a result. There were no episodes of mania or psychosis during any of the infusions in this trial. Although increases in blood pressure were more common in the ketamine group, these were transient and had returned to baseline during the post infusion monitoring period. Increases in blood pressure in the ketamine group became less prevalent with subsequent infusion sessions. The most common physical side-effects reported in the ketamine group were anxiety and dizziness, however these also lessened with subsequent infusions. Physical symptoms reported in this pilot trial are in keeping with those documented in previous trials ^19^. In the midazolam group, the most commonly reported symptom was fatigue, the prevalence of which was maintained as the treatment course continued. The vast majority of new onset physical symptoms in both groups were considered to be tolerable. Apart from palpitations reported in the second ketamine infusion by one participant and dizziness on standing reported in the second midazolam session, there were no other new onset symptoms that emerged during the treatment course after the first infusion session.

Previous studies have shown that a single infusion of subanaesthetic dose of ketamine has a fast acting antidepressant effect which is superior to placebo ^13^. This effect tends to start at 40 minutes after start of infusion, peak at day 1 and lose superiority at approximately day 10. Although this pilot trial was not sufficiently powered to detect differences in HRSD-24 depression scores, we did not observe the superior antidepressant effect of ketamine, with both groups having similar depression scores following each infusion period. No participants met response criteria within four hours of the first infusion session in either group. There have been very few trials that have examined serial ketamine infusions in depressed patients and results have been inconsistent in terms of its potential to prolong its antidepressant effects ^23– 25^. As this is a pilot trial with a small number of participants, we were unable to establish differences in depression scores at the follow-up time points. Further research is required to establish the possible sustained antidepressant effect of serial ketamine infusions.

Most previous trials that have used subanaesthetic doses of ketamine in the treatment of depression, have used saline as the placebo. There has been limited reporting of the success of blinding in many of these trials. In our pilot trial, 10/13 participants in the ketamine group correctly guessed their infusion and 7/12 in the midazolam group guessed correctly. Although these numbers are too small to interpret, it gives us some indication of the possible advantages of blinding when using midazolam as an active comparator. Midazolam mimics some of the psychoactive effects of ketamine making it potentially more acceptable as a placebo in clinical trials. A separate trial reported success of blinding in ketamine versus midazolam and had similar results to the Karma-Dep trial with 5/7 and 7/9 correctly guessing their allocated infusions to be ketamine and midazolam respectively ^42^. 

Strengths of this study include the use of midazolam as an active placebo, therefore, potentially helping with blinding and establishing an accurate treatment effect when compared to using only saline. Most other ketamine trials have used ketamine following a wash out period from other antidepressant therapies. As ketamine was used an as adjunctive treatment to other antidepressant medication and routine care in our trial, the results are more generalisable to real world practice.

 This trial has several limitations. As this is a pilot trial, it is not adequately powered to detect statistically significant differences between the depression scores of the two groups. Further limitations include eligibility being restricted to only inpatients within St Patrick’s University Hospital and participants not being permitted to continue with the infusion clinics if discharged early from hospital. We included those with both unipolar and bipolar depressive disorders but excluded those with a previous psychotic illness. Treatment as usual was continued for all participants by their treating teams which included medication changes and multidisciplinary team input which makes it difficult to distinguish between the antidepressant effects of ketamine and those of other medication changes and therapies.

## Conclusions

This study suggests that a definitive trial of adjunctive ketamine in the treatment of persons hospitalised for a depressive episode is feasible. Recruitment rates and retention rates were satisfactory. Ketamine and midazolam infusions were generally well tolerated with only a small proportion of participants (3/25, 12%) discontinuing treatment due to side-effects. More studies, however, are required to assess potential longer-term side-effects of repeated ketamine infusions.

## Data availability

### Underlying data

Access to raw data is restricted under Research Ethics Committees approval as even de-identified data may contain information that could potentially identify a participant which may present a potential breech in General Data Protection Regulation (GDPR). As a result, this data cannot be made available publicly. To access the data, please contact Bronagh Gallagher ( bgallag@tcd.ie) or the Principal Investigator ( d.mcloughlin@tcd.ie). Researchers must provide a written proposal on how the data will be used in research before access is granted.

### Extended data

Zenodo: Ketamine as an adjunctive therapy for major depression - a randomised controlled pragmatic pilot trial (Karma-Dep Trial). https://doi.org/10.5281/zenodo.4302297 ^43^

This file contains the following data:

- HPRA application protocol version 3.0 pdf.pdf (Original trial protocol)

### Reporting guidelines

Zenodo: CONSORT checklist for pilot studies for ‘Ketamine as an adjunctive therapy for major depression - a randomised controlled pragmatic pilot trial (Karma-Dep Trial)’ https://doi.org/10.5281/zenodo.4302297 ^43^

Data are available under the terms of the Creative Commons Attribution 4.0 International license (CC-BY 4.0).
