# Supplementary material for: Ketamine as an adjunctive therapy for major depression - a randomised controlled pragmatic pilot trial (Karma-Dep Trial)
Source: HRB Open Res. 2022 Jan 27;3:90. Originally published 2020 Dec 16. [Version 2] doi: 10.12688/hrbopenres.13182.2 (PMC8729188; doi:10.12688/hrbopenres.13182.2)
Supplement: Peer review history

# Peer review: Reviewer response for version 1

**DOI:** 10.21956/hrbopenres.14310.r31053

**Published:** 2022-01-04

**Authors:** Shane McInerney

**Affiliations:** 1 Department of Psychiatry, National University of Ireland, Galway, Ireland

This is an interesting pilot study examining the effect of intravenous ketamine compared to a placebo control on a depressed population of inpatients. The study is well designed and the paper is well written.

 The results did not find a statistically significiant difference in favour of ketamine. The sample size was acknowledged as being small and some patients were unable to tolerate the treatment. As patients were hospitalised in a private psychiatric hospital noted for its availability of a range of psychosocial treatments, the benefit of this additional care would need to be clarified in a larger study.

 Overall, the paper suggests the feasibility of a pragmatic trial of adjunctive serial ketamine infusions for hospitalised depression by this group.

Is the work clearly and accurately presented and does it cite the current literature?

Yes

If applicable, is the statistical analysis and its interpretation appropriate?

Yes

Are all the source data underlying the results available to ensure full reproducibility?

Yes

Is the study design appropriate and is the work technically sound?

Yes

Are the conclusions drawn adequately supported by the results?

Yes

Are sufficient details of methods and analysis provided to allow replication by others?

Yes

Reviewer Expertise:

Depression, cognition.

I confirm that I have read this submission and believe that I have an appropriate level of expertise to confirm that it is of an acceptable scientific standard.

# Peer review: Reviewer response for version 1

**DOI:** 10.21956/hrbopenres.14310.r28551

**Published:** 2020-12-21

**Authors:** George Kirov

**Affiliations:** 1 MRC Centre for Neuropsychiatric Genetics and Genomics, Cardiff University, Cardiff, UK

Gallagher et al report on a trial comparing 4 weekly infusions of ketamine v.s. midazolam for the treatment of patients hospitalised with a depressive episode. This is a pilot study, aiming mostly at finding out whether such a trial can be conducted on a larger scale. About 20% of eligible patients consented and the majority of them completed the study, therefore a larger trial is clearly feasible.

 The study raises a number of additional questions. The rapid antidepressant effect of single dose ketamine reported in the literature was not replicated, according to the authors, as there were no differences from the control substance. I think the truth is somewhere in the middle. The reduction in the HAMD scores were quite impressive for a population of hospitalised patients, who were mostly treatment resistant, and some of whom even required ECT. Midazolam is not a placebo and benzodiazepines are very effective drugs before tolerance develops. I am not surprised that depressed patients given infusions of midazolam would show improvements in symptoms, and some of these are possibly related to anxiety and sleep. The way the conclusions sound now gives an impression that i.v. ketamine had no effect whatsoever, which is not my take of these results. On the other hand, the ketamine group did not demonstrate what would be described as dramatic improvement in depression, as claimed by some authors, in fact the improvements are modest and the remission rates were only around 20-30%. These are actually the rates reported in other studies, so the current study, in my view, replicates previous results and gives a realistic picture of what to expect from ketamine infusions. This includes a very comprehensive account of side effects and the general management of such patients.

 One other suggestion is to cite the recent review by Marcantoni et al ^1^. (sorry, just out). This review lists more research projects than have been cited by the current authors. While there could be reasons they decided not to cite some of the additional papers, I felt that they should give a better account of the previous research, which is summarised by Marcantoni et al ^1^.

 In summary, this is a very clear study which gives a realistic view of the acute antidepressant effects of ketamine.

Is the work clearly and accurately presented and does it cite the current literature?

Partly

If applicable, is the statistical analysis and its interpretation appropriate?

Yes

Are all the source data underlying the results available to ensure full reproducibility?

Yes

Is the study design appropriate and is the work technically sound?

Yes

Are the conclusions drawn adequately supported by the results?

Yes

Are sufficient details of methods and analysis provided to allow replication by others?

Yes

Reviewer Expertise:

ECT; psychiatric genetics

I confirm that I have read this submission and believe that I have an appropriate level of expertise to confirm that it is of an acceptable scientific standard.

## References

[1] : A systematic review and meta-analysis of the efficacy of intravenous ketamine infusion for treatment resistant depression: January 2009 - January 2019. *J Affect Disord* .2020;277: 10.1016/j.jad.2020.09.007 831-841 10.1016/j.jad.2020.09.007 33065824
